# Urban-Rural Differences Explain the Association between Serum 25-Hydroxyvitamin D Level and Insulin Resistance in Korea

**DOI:** 10.3390/nu6125806

**Published:** 2014-12-11

**Authors:** Bo Mi Song, Yumie Rhee, Chang Oh Kim, Yoosik Youm, Kyoung Min Kim, Eun Young Lee, Ju-Mi Lee, Young Mi Yoon, Hyeon Chang Kim

**Affiliations:** 1 Department of Public Health, Yonsei University Graduate School, Seoul 120-752, Korea; E-Mail: sbm1396@yuhs.ac; 2 Cardiovascular and Metabolic Disease Etiology Research Center, Yonsei University College of Medicine, Seoul 120-752, Korea; E-Mails: jmlee01@yuhs.ac (J.-M.L.); yun3416@yuhs.ac (Y.M.Y.); 3 Department of Internal Medicine, Yonsei University College of Medicine, Seoul 120-752, Korea; E-Mails: yumie@yuhs.ac (Y.R.); cokim@yuhs.ac (C.O.K.); leyme@yuhs.ac (E.Y.L.); 4 Department of Sociology, Yonsei University College of Social Sciences, Seoul 120-752, Korea; E-Mail: yoosik@yonsei.ac.kr; 5 Department of Internal Medicine, Seoul National University Bundang Hospital, Seongnam 463-707, Korea; E-Mail: kyoungmin02@gmail.com; 6 Department of Preventive Medicine, Yonsei University College of Medicine, Seoul 120-752, Korea

**Keywords:** vitamin D, insulin resistance, elderly, Korean, residential area

## Abstract

An increasing number of studies report associations between low serum 25-hydroxyvitamin D [25(OH)D] level and insulin resistance; however, whether low vitamin D levels directly contribute to increased insulin resistance is unclear. We investigated the impact of residential area on the association between 25(OH)D and insulin resistance in elderly Koreans. Using data from the Korean Urban Rural Elderly study, we conducted cross-sectional analyses in 1628 participants (505 men and 1123 women). Serum 25(OH)D was analyzed as both continuous and categorized variables. Homeostasis model assessment for insulin resistance (HOMA-IR) was calculated using fasting blood glucose and insulin levels. In men, 25(OH)D level was inversely associated with HOMA-IR (standardized β = −0.133, *p* < 0.001) after adjustment for age, body mass index, waist circumference, smoking, alcohol intake, exercise, and study year. However, we noted significant urban-rural differences in 25(OH)D level (43.4* versus* 65.6 nmol/L; *p* < 0.001) and HOMA-IR (1.2* versus* 0.8 mmol·pmol/L^2^; *p* < 0.001). When we additionally adjusted for residential area, the association between 25(OH)D and HOMA-IR was attenuated (standardized β = −0.063, *p* = 0.115). In women, the association between 25(OH)D and HOMA-IR was not significant before or after adjustment for residential area. Environmental or lifestyle differences in urban and rural areas may largely explain the inverse association between serum 25(OH)D and insulin resistance.

## 1. Introduction

Vitamin D plays important roles in calcium and phosphate absorption in the intestine, sustaining sufficient concentrations thereof in the blood. Access to these minerals at bone-forming sites makes normal mineralization of bone possible [1,2]. Reports from across the world indicate that vitamin D deficiency is widespread and is re-emerging as a major health problem globally [3]. Studies from Asian countries, with a few exceptions, have reported a high prevalence of vitamin D deficiency in both sexes and all age groups [4,5,6,7,8,9,10].

Increasing evidence suggests that vitamin D deficiency may also be an important cause in a variety of nonskeletal disorders including impaired glucose metabolism [11,12,13,14]. Several studies have reported that low serum 25-hydroxyvitamin D [25(OH)D] may be significantly associated with increased insulin resistance [1,15,16]. On the other hand, other studies have failed to detect a significant relationship between circulating vitamin D levels and insulin resistance [17,18,19,20,21]. Therefore, whether or not low serum vitamin D concentrations directly contribute to the development of insulin resistance remains controversial.

In relation to differences in sunlight exposure and physical activity, residential area and one’s occupation can affect serum 25(OH)D levels, as well as insulin sensitivity. However, the impact of residential area or occupation on the association between serum 25(OH)D level and insulin resistance has not been appropriately assessed. Thus, we attempted to investigate the association between serum 25(OH)D and insulin resistance, as well as the impact of residential area thereon, in an elderly cohort recruited from urban and rural communities.

## 2. Experimental Section

### 2.1. Study Population

This study used data from the Korean Urban Rural Elderly (KURE) study, an ongoing community-based cohort study. The KURE study planned to recruit 4000 participants aged 65 years or older from urban and rural communities of South Korea. During the summer seasons (June to September) of 2012 and 2013, a total of 2025 participants from urban communities (Seodaemun-gu, Eunpyeong-gu, and Mapo-gu, Seoul, Korea) and rural communities (Yangsa-myeon and Hajeom-myeon, Gangwha-gun, Incheon, Korea) were enrolled. All participants completed a health questionnaire and health examinations following an identical protocol. The sampling and data collection procedures have been described in detail elsewhere [22]. We finally conducted a cross-sectional analysis in 1628 participants (505 men and 1123 women) aged 65 to 95 years old, after excluding those undergoing treatment with insulin injection or diabetes medication (*n* = 397). All participants provided written informed consent forms at the beginning of the study, which was approved by the Institutional Review Board of Severance Hospital at Yonsei University College of Medicine (approval number: 4-2012-0172; approval date: 3 May 2012).

### 2.2. Questionnaire Data

All participants were individually interviewed using standardized questionnaires to obtain information on their demographics, medical history, medication use, and health behaviors. Trained interviewers carried out the questionnaire surveys according to the predefined protocol, and double-checked whether responses were inappropriate or missing. Occupations were classified as managers; professionals; technicians and associated professionals; clerical support workers; service workers; sales workers; agricultural, forestry, and fishery workers; craft and related trades workers; plant and machine operators and assemblers; elementary workers; soldiers; housewives; and the unemployed, according to the Korea Standard Classification of Occupations [23]. Smoking status was classified into two groups: current smokers or current nonsmokers (past smokers or those who had never smoked). Alcohol intake was categorized as regular alcohol drinking or other (participants who drink less than once a week or not at all). Physical activity was investigated on a regular basis regardless of indoor or outdoor exercise and categorized as regular exercise or other.

### 2.3. Physical Examination

Study participants wore lightweight hospital gowns for convenient and reliable examinations. Standing height was measured up to 0.1 cm with a stadiometer (DS-102, JENIX, Seoul, Korea) and body weight was measured up to 0.1 kg with a digital scale (DB-150, CAS, Yangju, Korea) according to the pre-developed protocol. Body mass index (BMI) was calculated as weight in kilograms divided by the square of height in meters (kg/m^2^). Waist circumference was measured up to 0.1 cm at the midpoint between the lower borders of the rib cage and the iliac crest with an ergonomic circumference measuring tape (SECA 201, SECA, Hamburg, Germany). Participants were seated for at least five min before undergoing blood pressure measurement; two measurements at a five-min interval were obtained using an automatic sphygmomanometer (Omron HEM-7111, Omron Healthcare Co., Ltd., Kyoto, Japan). If the two measurements differed by ≥10 mmHg for either systolic or diastolic blood pressure, an extra measurement was conducted after five minutes, and the last two measurements were averaged for analyses.

### 2.4. Laboratory Assays

Overnight fasting blood samples from all participants were collected from the antecubital vein. Serum 25(OH)D, currently considered a reliable indicator of vitamin D store [24], was measured by an automated chemiluminescence immunoassay (Liaison, Diasorin, Dietzenbach, Germany). This assay is widely available and offers higher throughput capacity, lower sample volume requirement, and reduced operator error [25]. The intra-assay coefficient of variation thereof was 5.5% at 18.0 nmol/L and 4.8% at 319.5 nmol/L; the inter-assay coefficient of variation thereof was 12.9% at 18.0 nmol/L and 7.3% at 319.5 nmol/L. Fasting glucose level was measured using the colorimetry method. Fasting insulin level was measured in accordance with a chemiluminescence immunoassay, with an intra-assay coefficient of variation of 4.6% at 102.0 pmol/L and 3.3% at 864.7 pmol/L, and an inter-assay coefficient of variation of 5.9% at 102.0 pmol/L and 4.8% at 864.7 pmol/L. Insulin resistance was estimated by Homeostasis Model Assessment for Insulin Resistance (HOMA-IR), the product of fasting glucose level (mmol/L) and insulin level (pmol/L) divided by 135 [26].

### 2.5. Statistical Analysis

We evaluated differences in general characteristics and variables of interest between men and women. Continuous variables were described as mean and standard deviation (for normally distributed variables) or as median and interquartile range (for skewed variables), and tested by independent *t*-test and Wilcoxon rank sum test, respectively. Categorical variables were described as numbers (percentages) and tested by chi-square tests. General characteristics and concentrations of selected biomarkers were also analyzed according to three categories of serum 25(OH)D concentrations [2]. One-way analysis of variance was used for continuous variables and a chi-square test was used for categorical variables. As serum 25(OH)D, triglycerides, fasting glucose, insulin, and HOMA-IR were right-skewed, they were log-transformed for parametric tests. The relationships between serum 25(OH)D levels and other variables were evaluated using partial correlation coefficients while controlling for age. Multiple linear regression analyses were used to assess the independent association between serum 25(OH)D and HOMA-IR in three adjusted models: (1) adjusting for study year and age; (2) additional adjustment for BMI, waist circumference, smoking status, alcohol intake, and regular exercise; and (3) additional adjustment for residential area. All statistical analyses were performed using SAS version 9.2 (SAS Institute, Cary, NC, USA), and statistical significance was defined as a two-sided *p*-value less than 0.05.

## 3. Results

The general characteristics of the study participants are presented in Table 1. This study comprised 505 men with a mean age of 72.8 years and 1123 women with a mean age of 71.4 years. Mean BMI was significantly lower in men than in women; however, mean waist circumference was significantly higher in men than in women. The median serum 25(OH)D level was significantly higher in men than in women (49.4 *vs.* 39.9 nmol/L). Fasting glucose level was not significantly different between men and women. However, fasting insulin (29.4* vs.* 35.4 pmol/L) and HOMA-IR (1.1* vs.* 1.3 mmol·pmol/L^2^) were lower in men than in women. Cigarette smoking and alcohol drinking were more frequently reported in men than in women. Meanwhile, frequency of regular exercise was similar between men and women.

**Table 1:** General characteristics of the study participants.

Variables	Men (*n* = 505)	Women (*n* = 1123)	*p*-value
Age, year	72.8 ± 4.9	71.4 ± 4.7	<0.001
Height, cm	164.6 ± 5.4	152.2 ± 5.6	<0.001
Weight, kg	64.3 ± 8.9	56.3 ± 8.0	<0.001
Body mass index, kg/m^2^	23.7 ± 2.9	24.3 ± 3.0	<0.001
Waist circumference, cm	85.7 ± 8.6	82.7 ± 8.7	<0.001
Systolic blood pressure, mmHg	129.5 ± 14.5	127.4 ± 15.7	0.008
Diastolic blood pressure, mmHg	74.1 ± 8.8	72.7 ± 8.6	0.004
Total cholesterol, mmol/L	4.5 ± 0.8	5.0 ± 0.9	<0.001
HDL cholesterol, mmol/L	1.2 ± 0.3	1.4 ± 0.3	<0.001
LDL cholesterol, mmol/L	2.7 ± 0.7	2.9 ± 0.8	<0.001
Triglycerides, mmol/L	1.3 [0.3–1.7]	1.3 [1.0–1.8]	0.040
25(OH)D, nmol/L	49.4 [35.4–61.9]	39.9 [28.2–54.9]	<0.001
Fasting glucose, mmol/L	5.1 [4.8–5.5]	5.1 [4.8–5.4]	0.364
Fasting insulin, pmol/L	29.4 [19.8–43.8]	35.4 [24.6–53.4]	<0.001
HOMA-IR, mmol·pmol/L^2^	1.1 [0.7–1.7]	1.3 [0.9–2.1]	<0.001
HOMA-IR ≥ 2.5, *n* (%)	56 (11.1)	190 (16.9)	0.003
Impaired fasting glucose, *n* (%)	116 (23.0)	218 (19.4)	0.115
Hypertension, *n* (%)	293 (58.0)	694 (61.8)	0.165
Dyslipidemia, *n* (%)	243 (48.1)	627 (55.8)	0.005
Current smoker, *n* (%)	81 (16.0)	17 (1.5)	<0.001
Regular alcohol drinker, *n* (%)	195 (38.6)	58 (5.2)	<0.001
Regular exercise, *n* (%)	282 (55.8)	663 (59.0)	0.248

Data are expressed as mean ± standard deviation, median [inter quartile range], or number (percent). LDL cholesterol levels were calculated for 499 men and 1119 women. Abbreviations: HDL, high-density lipoprotein; LDL, low-density lipoprotein; HOMA-IR, homeostasis model assessment for insulin resistance.

Table 2 presents the characteristics of the study population according to 25(OH)D concentration: <50 (deficient), 50 to 75 (insufficient), and ≥75 nmol/L (sufficient) [2]. The majority of the participants (52% of men and 69% of women) were 25(OH)D deficient, while an additional 36% of men and 25% of women were 25(OH)D insufficient. Men and women with higher 25(OH)D concentrations tended to have lower BMI and waist circumference. Participants with higher 25(OH)D concentrations were also associated with lower fasting insulin and HOMA-IR levels, although the association was statistically significant only in men. Table 3 presents the linear correlations between serum 25(OH)D and indices of obesity and glucose metabolism. After controlling for age, serum 25(OH)D level was negatively correlated with fasting insulin and HOMA-IR but not with fasting glucose.

**Table 2:** Characteristics of the study participants by vitamin D concentration.

Variables	Men (*n* = 505)				Women (*n* = 1123)			
	<50 (*n* = 264, 52.3%)	50–75 (*n* = 184, 36.4%)	≥75 (*n* = 57, 11.3%)	*p* for Trend	<50 (*n* = 771, 68.7%)	50–75 (*n* = 280, 24.9%)	≥75 (*n* = 72, 6.4%)	*p* for Trend
Age, year	72.7 ± 4.8	72.9 ± 4.7	72.8 ± 5.7	0.937	71.5 ± 4.7	71.2 ± 4.5	71.6 ± 4.9	0.907
Height, cm	164.4 ± 5.4	165.0 ± 5.2	163.9 ± 6.1	0.517	152.2 ± 5.6	152.2 ± 5.8	152.5 ± 4.8	0.625
Weight, kg	64.8 ± 8.4	64.4 ± 9.5	61.8 ± 9.1	0.021	56.8 ± 8.2	55.4 ± 7.6	54.3 ± 7.2	0.012
Body mass index, kg/m^2^	24.0 ± 2.8	23.6 ± 3.0	22.9 ± 2.6	0.014	24.5 ± 3.1	23.9 ± 2.8	23.4 ± 2.9	0.002
Waist circumference, cm	86.3 ± 8.4	85.5 ± 8.8	83.3 ± 8.5	0.017	83.2 ± 8.8	81.9 ± 8.4	79.9 ± 7.6	0.002
Systolic BP, mmHg	129.1 ± 15.9	130.2 ± 13.1	129.2 ± 11.3	0.978	127.7 ± 16.1	127.2 ± 14.6	125.7 ± 15.7	0.316
Diastolic BP, mmHg	74.0 ± 9.4	74.1 ± 8.1	74.3 ± 8.5	0.867	72.8 ± 9.0	72.7 ± 8.1	71.8 ± 7.4	0.350
Total cholesterol, mmol/L	4.63 ± 0.83	4.48 ± 0.82	4.35 ± 0.71	0.019	5.04 ± 0.92	4.82 ± 0.93	4.56 ± 0.83	<0.001
HDL cholesterol, mmol/L	1.22 ± 0.33	1.25 ± 0.29	1.32 ± 0.33	0.038	1.35 ± 0.34	1.38 ± 0.33	1.34 ± 0.32	0.688
LDL cholesterol, mmol/L	2.72 ± 0.76	2.61 ± 0.72	2.47 ± 0.66	0.019	2.99 ± 0.82	2.81 ± 0.81	2.62 ± 0.71	<0.001
Triglycerides, mmol/L	116 [84–161]	103 [79–152]	102 [90–123]	0.021	120 [90–165]	113 [82–152]	102 [76–140]	0.014
Fasting glucose, mmol/L	91 [86–99]	91 [86–98]	92 [87–98]	0.596	91 [86–98]	90 [85–95]	90 [86–97]	0.471
Fasting insulin, pmol/L	5.3 [3.7–7.9]	4.7 [3.2–7.1]	3.5 [2.5–5.1]	<0.001	6.2 [4.3–9.2]	5.2 [3.7–8.4]	5.5 [4.2–7.6]	0.059
HOMA-IR, mmol·pmol/L^2^	1.20 [0.80–1.88]	1.04 [0.71–1.66]	0.83 [0.56–1.30]	<0.001	1.41 [0.97–2.16]	1.20 [0.79–1.93]	1.24 [0.92–1.80]	0.060
HOMA-IR ≥ 2.5, *n* (%)	36 (13.6)	19 (10.3)	1 (1.8)	0.013 *	140 (18.2)	42 (15.0)	8 (11.1)	0.070 *
Current smoker, *n* (%)	44 (16.7)	29 (15.8)	8 (14.0)	0.620 *	13 (1.7)	3 (1.1)	1 (1.4)	0.565 *
Regular alcohol drinker, *n* (%)	88 (33.3)	78 (42.4)	29 (50.9)	0.005 *	49 (6.4)	8 (2.9)	1 (1.4)	0.008 *
Regular exercise, *n* (%)	166 (62.9)	91 (49.5)	25 (43.9)	<0.001 *	459 (59.5)	161 (57.5)	43 (59.7)	0.738 *

Data are expressed as mean ± standard deviation, median [inter quartile range], or number (percent). *p* for trend was derived from a general linear model using contrast coefficients or from the Cochran-Armitage trend test *. Abbreviations: BP, blood pressure; HDL, high-density lipoprotein; LDL, low-density lipoprotein; HOMA-IR, homeostasis model assessment for insulin resistance.

**Table 3:** Pearson’s correlation coefficients between serum 25(OH)D * and other variables.

Variables	Men (*n* = 505)		Women (*n* = 1123)	
	Partial Correlation Coefficient ^†^	*p*-value	Partial Correlation Coefficient ^†^	*p*-value
Body mass index	−0.152	0.005	−0.126	<0.001
Waist circumference	−0.117	0.009	−0.107	<0.001
Fasting glucose *	−0.071	0.110	−0.035	0.245
Fasting insulin *	−0.211	<0.001	−0.096	0.001
HOMA-IR *	−0.207	<0.001	−0.095	0.001

* Analyzed with log-transformed values. ^†^ Age-adjusted. Abbreviation: HOMA-IR, homeostasis model assessment for insulin resistance.

We compared the distributions of serum 25(OH)D level and HOMA-IR according to residential area (Table 4). Urban-living male participants exhibited lower serum 25(OH)D levels (43.4* vs.* 65.6 nmol/L) but higher HOMA-IR (1.2* vs.* 0.8 mmol·pmol/L^2^), compared to their rural-living counterparts. Similarly, urban-living female participants had lower serum 25(OH)D levels (38.2* vs.* 49.9 nmol/L) but higher HOMA-IR (1.4* vs.* 1.2 mmol·pmol/L^2^), compared to their rural-living counterparts.

**Table 4:** Serum 25(OH)D and HOMA-IR according to residential area.

Region	Men (*n* = 505)		Region	Women (*n* = 1123)	
	25(OH)D	HOMA-IR		25(OH)D	HOMA-IR
Urban (*n* = 371)	43.4 [32.7–55.2]	1.2 [0.8–1.8]	Urban (*n* = 948)	38.2 [27.2–51.9]	1.4 [0.9–2.1]
Rural (*n* = 134)	65.6 [54.2–76.1]	0.8 [0.5–1.3]	Rural (*n* = 175)	49.9 [36.7–61.7]	1.2 [0.8–1.8]
*p*-value	<0.001	<0.001	*p*-value	<0.001	0.002

Data are expressed as median [inter quartile range]. Abbreviation: HOMA-IR, homeostasis model assessment for insulin resistance.

Table 5 presents the association between serum 25(OH)D level and HOMA-IR after multiple linear regression analyses. In men, serum 25(OH)D levels showed a significant inverse association with HOMA-IR (standardized β = −0.203, *p* < 0.001) when adjusted for age and study year. The association remained significant (standardized β = −0.133, *p* < 0.001) after additionally adjusting for BMI, waist circumference, smoking, alcohol intake, and exercise. However, the association was markedly attenuated after additional adjustment for residential area (standardized β = −0.063, *p* = 0.115). In women, serum 25(OH)D was significantly associated with HOMA-IR (standardized β = −0.092, *p* < 0.001) when adjusted for age and study year. However, the association disappeared after additional adjustment for BMI, waist circumference, smoking, alcohol intake, and exercise (*p* = 0.187). When we additionally assessed the association between serum 25(OH)D and HOMA-IR, stratified by residential area, we found no significant association for either urban or rural participants (Table 6). When we controlled occupation instead of residential area, similar results were observed (data presented in Supplemental Table 1 and 2). However, we did not differentiate the effects of residential area and occupation, because they are too closely correlated.

**Table 5:** Association between log-transformed serum 25(OH)D and log-transformed HOMA-IR in men and women.

Variables	Men (*n* = 505)						Women (*n* = 1123)					
	std. β	*p*-value	std. β	*p*-value	std. β	*p*-value	std. β	*p*-value	std. β	*p*-value	std. β	*p*-value
25(OH)D, nmol/L	–0.203	<0.001	–0.133	<0.001	−0.063	0.115	–0.092	<0.001	–0.035	0.187	–0.022	0.415
Study year, year	0.057	0.193	0.054	0.148	0.067	0.067	0.057	0.055	0.074	0.007	0.076	0.006
Age, year	–0.018	0.689	0.042	0.264	0.056	0.130	0.031	0.301	0.026	0.341	0.035	0.201
Body mass index, kg/m^2^			0.318	<0.001	0.321	<0.001			0.257	<0.001	0.264	<0.001
Waist circumference, cm			0.249	<0.001	0.225	<0.001			0.231	<0.001	0.222	<0.001
Current smoker (*vs.* others)			–0.074	0.045	–0.071	0.049			–0.006	0.825	–0.008	0.751
Regular alcohol drinker (*vs.* others)			–0.001	0.981	0.001	0.978			–0.010	0.718	–0.011	0.685
Regular exercise (*vs.* none)			0.027	0.468	–0.012	0.751			–0.003	0.920	–0.021	0.456
Rural (*vs.* urban)					–0.183	<0.001					–0.074	0.008
Coefficient of determination	adj. *R*^2^ = 0.041		adj. *R*^2^ = 0.336		adj. *R*^2^ = 0.360		adj. *R*^2^ = 0.011		adj. *R*^2^ = 0.219		adj. *R*^2^ = 0.223	

Abbreviation: HOMA-IR, homeostasis model assessment for insulin resistance.

**Table 6:** Association between log-transformed serum 25(OH)D and log-transformed HOMA-IR in men and women according to residential area.

Variables	Urban (*n* = 1319)				Rural (*n* = 309)			
	Men (*n* = 371)		Women (*n* = 948)		Men (*n* = 134)		Women (*n* = 175)	
	std. β	*p*-value	std. β	*p*-value	std. β	*p*-value	std. β	*p*-value
25(OH)D, nmol/L	−0.052	0.263	−0.008	0.785	−0.084	0.217	−0.084	0.224
Study year, year	0.101	0.035	0.046	0.129	0.035	0.582	0.180	0.011
Age, year	0.141	0.003	0.060	0.044	−0.120	0.090	−0.084	0.236
Body mass index, kg/m^2^	0.281	<0.001	0.304	<0.001	0.422	0.002	0.116	0.308
Waist circumference, cm	0.224	0.003	0.178	<0.001	0.221	0.094	0.369	0.001
Current smoker (*vs.* others)	−0.054	0.240	−0.003	0.910	−0.114	0.091	−0.072	0.285
Regular alcohol drinker (*vs.* others)	0.037	0.433	−0.021	0.464	−0.105	0.120	0.080	0.231
Regular exercise (*vs.* none)	−0.033	0.480	−0.010	0.747	0.007	0.918	−0.018	0.790
Coefficient of determination	adj. *R*^2^ = 0.241		adj. *R*^2^ = 0.215		adj. *R*^2^ = 0.467		adj. *R*^2 ^= 0.250	

Abbreviation: HOMA-IR, homeostasis model assessment for insulin resistance.

## 4. Discussion

The current study was designed to examine the impact of residential area or occupation on the association between serum 25(OH)D level and insulin resistance. Herein, we observed a significant association between serum 25(OH)D and HOMA-IR in men; however, the association was markedly attenuated after adjusting for residential area or occupation.

Several studies, including a few reports from the Korean population, have revealed inverse associations between vitamin D and insulin resistance [1,15,16,27,28]. A random sample of the general population of Copenhagen, Denmark, demonstrated that low 25(OH)D level is not significantly related to incident type 2 diabetes mellitus after adjusting for confounders; however, it was significantly associated with adverse longitudinal changes in continuous markers of glucose homeostasis [27]. In a nested case-control study performed on US military service members, participants with a low serum 25(OH)D level exhibited a substantially higher risk of developing insulin-requiring diabetes mellitus than those with a higher level [15]. In a Thai population study, low vitamin D level was shown to be modestly associated with a small increase in risk of type 2 diabetes mellitus in urban elderly residents only [28]. In the Fourth Korea National Health and Nutrition Examination Survey (KNHANES), a low serum 25(OH)D level was associated with fasting insulin, HOMA-IR, and diabetes [1,16].

Meanwhile, other studies have shown that serum 25(OH)D level is not significantly correlated to glucose metabolism [17,18,19,20,21,29]. Our previous study suggested that vitamin D is not independently associated with insulin resistance among middle-aged Korean men and women [29]. In the Third U.S. National Health and Nutrition Examination Survey, serum 25(OH)D level was inversely associated with insulin resistance and diabetes mellitus only in Mexican Americans and non-Hispanic whites, but not in non-Hispanic blacks [17]. In the Hoorn study, which comprised participants aged 50 to 75 years, no significant relationship was revealed between 25(OH)D, postprandial or fasting glucose concentrations, and incident diabetes [18]. In a healthy Cree community in Quebec, Canada, no association between vitamin D and insulin homeostasis indices (HOMA-IR and HOMA-Beta) was detected [19]. In a cross-sectional study of Pan-European subjects with metabolic syndrome, no correlations were recorded between vitamin D and intravenous glucose tolerance test (IVGTT)-based estimates of insulin secretion and action [20]. Additionally, a Turkish study of children and adolescents found no correlation between insulin measurements during an oral glucose tolerance test and vitamin D deficiency [21].

Compared with previous studies, ours is distinct in that we investigated the gender and residential area-specific association between low 25(OH)D level and insulin resistance in general elderly Koreans, while other Korean studies were carried out indiscriminately on all participants aged 19 years or older with statistical adjustments for gender and residential area [1,16]. Previous studies that set out to investigate the inverse association between vitamin D and insulin resistance also might not have appropriately controlled for residential area and other socio-demographic characteristics. In fact, many previous studies categorized residential areas into rural and urban areas simply based on administrative district, which may not reflect actual urban-rural differences.

The KURE study recruited participants from urban and rural areas. All participants from the two areas resided in the midwestern region of the Korean peninsula, and were of the same ethnic and racial origin, Korean. Total cholesterol, HDL cholesterol, LDL cholesterol, and triglyceride levels were similar between the urban dwellers and the rural dwellers. Distribution of smoking status and alcohol intake were also similar between the two communities. However, there were some differences between the urban and rural residents: in the urban area, only 0.1% of participants worked in the agricultural, forestry, or fishery industry, whereas in the rural area, 60.8% of participants were agricultural, forestry, or fishery workers; people working in these industries typically spend a lot more time working outdoors. Also, urban dwellers were more obese than the rural dwellers (BMI, 24.2* vs.* 23.5 kg/m^2^, *p* < 0.001; waist circumference, 84.0* vs.* 82.1 cm,* p* < 0.001). Thus, it was possible for us to take into account residential area as a potential confounder. To compare the effect of residential area* versus* the effect of individual characteristics on the association between serum 25(OH)D and insulin resistance in men, we constructed an additional model that included adjustment for residential area in addition to study year and a participant’s age. Adjusting for residential area resulted in greater attenuation of the effect size (std. β = −0.100, *p* = 0.035) than adjusting for individual characteristics, such as BMI, waist circumference, smoking, alcohol intake, and exercise (std. β = −0.133, *p* < 0.001). These results implied that urban-rural differences exert a greater influence on the association between serum 25(OH)D and insulin resistance than other measurable variables: we suspect that urban-rural differences are reflective of any number of combinations of environmental and socioeconomic factors, such as dissimilarities in outdoor activity related to one’s occupation and physical activity related to traveling to and from work. Nonetheless, our findings suggest that vitamin D itself does not exert major influences on insulin resistance and that residential area largely explains the association between 25(OH)D and insulin resistance.

The study has a few limitations that warrant consideration. First, this study was conducted as a cross-sectional study in which all information was collected at the same point in time; therefore, the causal association between serum 25(OH)D and insulin resistance is uncertain and residual confounders might not be totally removed. Second, we did not examine intake of vitamin D supplements and outdoor activity, which may influence vitamin D concentrations in the body, and thus we could not adjust for them. Third, we did not utilize the euglycemic clamp method, known as a gold standard examination for assessing insulin resistance. Instead, we utilized HOMA-IR as a surrogate marker of insulin resistance. Most epidemiological studies widely support the use of HOMA-IR on the basis of a high correlation between estimates of insulin resistance derived from HOMA and from the glucose clamp [30]. Lastly, serum 25(OH)D level was not measured by tandem-mass spectrometry, the reference method, but by chemiluminescence immunoassay. This immunoassay is intended for quantitative determination of total 25(OH)D and does not distinguish between 25(OH)D_2_ and 25(OH)D_3_ [31]. Also, serum 25(OH)D level was measured mainly in the summer and cannot represent annual mean concentrations. Nevertheless, the principal purpose of the current study was to examine the relationship between serum 25(OH)D and insulin resistance in a general elderly population and not to report absolute levels of 25(OH)D over the year. Thus, our findings would not be critically distorted by the single measurement of serum 25(OH)D levels.

## 5. Conclusions

In conclusion, lower concentrations of vitamin D are associated with increased insulin resistance in elderly Korean men. However, the inverse association between serum 25(OH)D and insulin resistance was largely explained by environmental or lifestyle differences in urban and rural areas. Our findings do not support a causal relationship between serum 25(OH)D and type 2 diabetes, although they cannot rule out a weak causal effect.

## Supplementary Files

Supplementary File 1
