# Supplementary material for: Urban-Rural Differences Explain the Association between Serum 25-Hydroxyvitamin D Level and Insulin Resistance in Korea
Source: Nutrients. 2014 Dec 11;6(12):5806–18. doi: 10.3390/nu6125806 (PMC4277000; doi:10.3390/nu6125806)
Supplement: Supplementary File 1 [file nutrients-06-05806-s001.docx]

**Supplementary Information**

**Table S1.** Association between log-transformed serum 25(OH)D and log-transformed HOMA-IR in men and women.

| **Variables** | **Men (*n* = 505)** | | | | | | **Women (*n* = 1123)** | | | | | |
| --- | --- | --- | --- | --- | --- | --- | --- | --- | --- | --- | --- | --- |
|  | **std. β** | ***p*-value** | **std. β** | ***p*-value** | **std. β** | ***p*-value** | **std. β** | ***p*-value** | **std. β** | ***p*-value** | **std. β** | ***p*-value** |
| 25(OH)D, nmol/L | −0.203 | <0.001 | −0.133 | <0.001 | −0.067 | 0.088 | −0.092 | <0.001 | −0.035 | 0.187 | −0.027 | 0.311 |
| Study year, year | 0.057 | 0.193 | 0.054 | 0.148 | 0.058 | 0.115 | 0.057 | 0.055 | 0.074 | 0.007 | 0.075 | 0.006 |
| Age, year | −0.018 | 0.689 | 0.042 | 0.264 | 0.046 | 0.213 | 0.031 | 0.301 | 0.026 | 0.341 | 0.028 | 0.295 |
| Body mass index, kg/m^2^ |  |  | 0.318 | <0.001 | 0.319 | <0.001 |  |  | 0.257 | <0.001 | 0.263 | <0.001 |
| Waist circumference, cm |  |  | 0.249 | <0.001 | 0.221 | <0.001 |  |  | 0.231 | <0.001 | 0.223 | <0.001 |
| Current smoker (*vs.* others) |  |  | −0.074 | 0.045 | −0.070 | 0.054 |  |  | −0.006 | 0.825 | −0.008 | 0.760 |
| Regular alcohol drinker (*vs.* others) |  |  | −0.001 | 0.981 | 0.001 | 0.988 |  |  | −0.010 | 0.718 | −0.010 | 0.702 |
| Regular exercise (*vs.* no) |  |  | 0.027 | 0.468 | −0.006 | 0.865 |  |  | −0.003 | 0.920 | −0.013 | 0.631 |
| Agricultural, forestry, and fishery workers (*vs.* others) |  |  |  |  | −0.181 | <0.001 |  |  |  |  | −0.058 | 0.033 |
| Coefficient of determination | adj. *R*^2^ = 0.041 | | adj. *R*^2^ = 0.336 | | adj. *R*^2^ = 0.361 | | adj. *R*^2^ = 0.012 | | adj. *R*^2^ = 0.219 | | adj. *R*^2^ = 0.221 | |

Abbreviation: HOMA-IR, homeostasis model assessment for insulin resistance.

**Table S2.** Association between log-transformed serum 25(OH)D and log-transformed HOMA-IR in men and women according to occupation.

| **Variables** | **Others (*n* = 1424)** | | | | **Agricultural, forestry,  and fishery workers (*n* = 204)** | | | |
| --- | --- | --- | --- | --- | --- | --- | --- | --- |
|  | **Men (*n* = 402)** | | **Women (*n* = 1022)** | | **Men (*n* = 103)** | | **Women (*n* = 101)** | |
|  | **std. β** | ***p*-value** | **std. β** | ***p*-value** | **std. β** | ***p*-value** | **std. β** | ***p*-value** |
| 25(OH)D, nmol/L | −0.065 | 0.142 | −0.018 | 0.514 | −0.065 | 0.384 | −0.108 | 0.246 |
| Study year, year | 0.048 | 0.286 | 0.057 | 0.050 | 0.100 | 0.182 | 0.233 | 0.012 |
| Age, year | 0.068 | 0.131 | 0.032 | 0.256 | 0.002 | 0.975 | −0.031 | 0.733 |
| Body mass index, kg/m^2^ | 0.279 | <0.001 | 0.272 | <0.001 | 0.486 | 0.001 | 0.169 | 0.269 |
| Waist circumference, cm | 0.236 | 0.002 | 0.214 | <0.001 | 0.192 | 0.164 | 0.260 | 0.081 |
| Current smoker (*vs.* others) | −0.060 | 0.177 | −0.008 | 0.785 | −0.107 | 0.167 | NA | |
| Regular alcohol drinker (*vs.* others) | 0.020 | 0.663 | −0.023 | 0.416 | −0.081 | 0.293 | 0.197 | 0.036 |
| Regular exercise (*vs.* no) | −0.003 | 0.947 | −0.007 | 0.803 | −0.002 | 0.978 | 0.036 | 0.691 |
| Coefficient of determination | adj. *R*^2^ = 0.242 | | adj. *R*^2^ = 0.214 | | adj. *R*^2^ = 0.461 | | adj. *R*^2^ = 0.265 | |

Abbreviation: HOMA-IR, homeostasis model assessment for insulin resistance.

© 2014 by the authors; licensee MDPI, Basel, Switzerland. This article is an open access article distributed under the terms and conditions of the Creative Commons Attribution license (http://creativecommons.org/licenses/by/4.0/).
